# Carbon source regulates polysaccharide capsule biosynthesis in *Streptococcus pneumoniae*

**DOI:** 10.1074/jbc.RA119.010764

**Published:** 2019-10-08

**Authors:** Lukas J. Troxler, Joel P. Werren, Thierry O. Schaffner, Nadezda Mostacci, Peter Vermathen, Martina Vermathen, Daniel Wüthrich, Cedric Simillion, Silvio D. Brugger, Rémy Bruggmann, Lucy J. Hathaway, Julien Furrer, Markus Hilty

**Affiliations:** a Institute for Infectious Diseases, Faculty of Medicine, University of Bern, 3001 Bern, Switzerland; b Graduate School for Cellular and Biomedical Sciences, University of Bern, 3012 Bern, Switzerland; c Department of BioMedical Research and Radiology, University of Bern and Inselspital, 3012 Bern, Switzerland; d Department of Chemistry and Biochemistry, University of Bern, 3012 Bern, Switzerland; e Interfaculty Bioinformatics Unit and Swiss Institute of Bioinformatics, University of Bern, 3012 Bern, Switzerland; f Applied Microbiology Research Unit, Department of Biomedicine, University of Basel, 4031 Basel, Switzerland; g Division of Clinical Microbiology, University Hospital Basel, 4031 Basel, Switzerland; h Department of Infectious Diseases and Hospital Epidemiology, University Hospital Zurich, University of Zurich, 8091 Zurich, Switzerland; i The Forsyth Institute (Microbiology), Cambridge, Massachusetts 02142; j Department of Oral Medicine, Infection and Immunity, Harvard School of Dental Medicine, Boston, Massachusetts 02142

**Keywords:** fructose, Streptococcus, nuclear magnetic resonance (NMR), bacterial metabolism, glucose, virulence factor, carbohydrate metabolism, exopolysaccharide capsule, serotype, Streptococcus pneumoniae, sucrose

## Abstract

The exopolysaccharide capsule of *Streptococcus pneumoniae* is an important virulence factor, but the mechanisms that regulate capsule thickness are not fully understood. Here, we investigated the effects of various exogenously supplied carbohydrates on capsule production and gene expression in several pneumococcal serotypes. Microscopy analyses indicated a near absence of the capsular polysaccharide (CPS) when *S. pneumoniae* was grown on fructose. Moreover, serotype 7F pneumococci produced much less CPS than strains of other serotypes (6B, 6C, 9V, 15, and 23F) when grown on glucose or sucrose. RNA-sequencing revealed carbon source-dependent regulation of distinct genes of WT strains and capsule-switch mutants of serotypes 6B and 7F, but could not explain the mechanism of capsule thickness regulation. In contrast, ^31^P NMR of whole-cell extract from capsule-knockout strains (Δ*cps*) clearly revealed the accumulation or absence of capsule precursor metabolites when cells were grown on glucose or fructose, respectively. This finding suggests that fructose uptake mainly results in intracellular fructose 1-phosphate, which is not converted to CPS precursors. In addition, serotype 7F strains accumulated more precursors than did 6B strains, indicating less efficient conversion of precursor metabolites into the CPS in 7F, in line with its thinner capsule. Finally, isotopologue sucrose labeling and NMR analyses revealed that the uptake of the labeled fructose subunit into the capsule is <10% that of glucose. Our findings on the effects of carbon sources on CPS production in different *S. pneumoniae* serotypes may contribute to a better understanding of pneumococcal diseases and could inform future therapeutic approaches.

## Introduction

**Figure 1.**
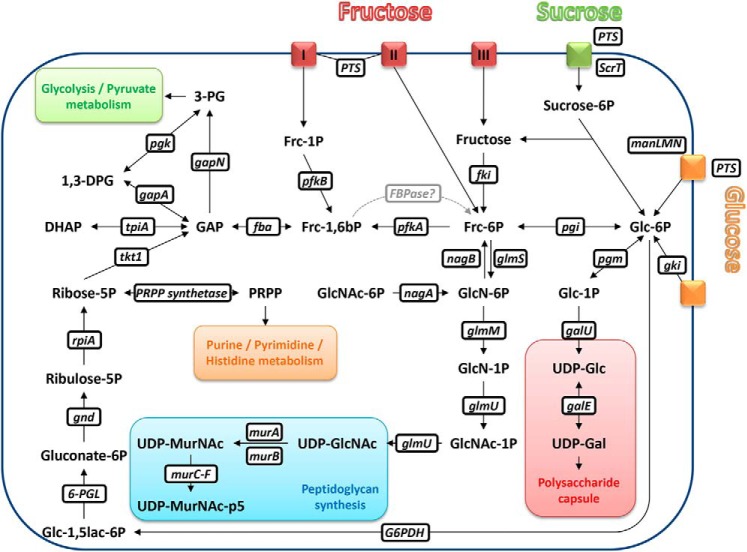
**Schematic illustration of metabolic pathways in *S. pneumoniae*.** Adapted from Refs. 3, 11, 14, and 53. *I*: *fruA* fructose uptake phosphotransferase transporter (*red*); *II*: *manLMN* monosaccharide uptake phosphotransferase transporter (*red*); *III*: membrane-spanning protein able to take up free fructose, PtsG analog (*red*); *1,3-DPG*, 1,3-diphosphoglycerate; *3-PG*, 3-phosphoglycerate; *6-PGL*, 6-phosphogluconolactonase; *DHAP*, dihydroxyacetone phosphate; *fba,* fructose-bisphosphate aldolase; *fki,* fructokinase; *Frc-1,6bP*, fructose 1,6-bisphosphate; *Frc-1P*, fructose 1-phosphate; *Frc-6P*, fructose 6-phosphate; *G6PDH*, glucose 6-phosphate dehydrogenase; *gale,* UDP-glucose 4-epimerase; *galU,* glucose 1-phosphate uridyltransferase; GAP, glyceraldehyde 3-phosphate; *gapA,* glyceraldehyde 3-phosphate dehydrogenase; *gapN,* glyceraldehyde 3-phosphate dehydrogenase (NADP+); *gki,* glucokinase (*orange*); *Glc-1,5lac-6P*, glucono-1,5-lactone 6-phosphate; *Glc-1P*, glucose 1-phosphate; *Glc-6P*, glucose 6-phosphate; *GlcN-1P*, glucosamine 1-phosphate; *GlcN-6P*, glucosamine 6-phosphate; *GlcNAc-1P*, GlcNAc 1-phosphate; *GlcNAc-6P*, GlcNAc 6-phosphate; *glmM,* phosphoglucosamine mutase; *glmS,* glutamine-fructose 6-phosphate aminotransferase; *glmU,* bifunctional protein (acetyltransferase, uridyltransferase); *gnd,* 6-phosphogluconate dehydrogenase; *manLMN,* monosaccharide uptake phosphotransferase (*orange*); *murA,* UDP-GlcNAc enolpyruvyl transferase; *murB,* UDP-*N*-acetylenolpyruvoylglucosamine reductase; *murC-F,* peptide ligases; *nagA,* α-*N*-acetylgalactosaminidase; *nagB,* glucosamine 6-phosphate deaminase; *pfkA,* ATP-dependent 6-phosphofructokinase; *pfkB,* 1-phosphofructokinase; *pgi,* glucose 6-phosphate isomerase; *pgk,* phosphoglycerate kinase; *pgm,* phosphoglucomutase; *rpiA,* ribose-5-phosphate isomerase; *ScrT,* sucrose phosphotransferase transporter (*green*); *tkt1,* transketolase; *tpiA,* triose-phosphate isomerase; *UDP-Gal*, uridine diphosphate galactose; *UDP-Glc*, uridine diphosphate glucose; *UDP-GlcNAc*, uridine diphosphate GlcNAc; *UDP-MurNAc*, uridine diphosphate *N*-acetylmuramate; *UDP-MurNAc-p5*, uridine diphosphate *N*-acetylmuramate pentapeptide. Hypothetical fructose-1,6-bisphosphatase (*FBPase*) is indicated in *gray*.

*Streptococcus pneumoniae* (the pneumococcus) is a pathobiont mainly colonizing the nasal passages but capable of causing invasive disease (1). Thus it is exposed to a variety of nutrient profiles in its environment, such as a glucose-dominated environment in the blood or a galactose (and GlcNAc)-dominated environment in the respiratory mucus of the nasopharynx (2). Recent studies have investigated the host glycan sugar-specific pathways in *S. pneumoniae* (3, 4). This has provided an accurate map of the biochemical pathways for the pneumococcal galactose, mannose, and GlcNAc catabolism. It also has been shown that the transcriptional response to glucose is strong and that in the central carbon metabolism, glucose exerts mostly negative regulation (5). Recently, the growth and metabolism of *S. pneumoniae* has been studied by *in vivo* NMR techniques, which has paved the way to a better understanding of central metabolism regulation (6) (Fig. 1). The central carbon metabolism of *S. pneumoniae* has also been studied by isotopologue profiling, which allowed investigation of the biosynthesis of amino acids (7).

It is believed that the nutritional environment may not only influence the metabolism but is also relevant for the expression of one of the main pneumococcal virulence factors, its polysaccharide capsule. It has been shown that changes in availability of oxygen accentuate differences in capsular polysaccharide expression (8). In addition, we have studied the capsule type in pneumococcal strains grown in nutrient-restricted Lacks medium (MLM) and in rich undefined brain heart infusion broth supplemented with 5% fetal calf serum (BHI + FCS) and have shown that certain pneumococcal strains produce less exopolysaccharide if grown in nutrient-restricted conditions (9). Different carbon sources present in the environment may also affect capsule expression differently. Using chemically defined media (CDM),^2^ it has been shown that *S. pneumoniae* grown on fructose produce less capsule polysaccharide than those grown on glucose as the sole carbon source (10). The effect of other carbon sources on the capsule is less clear, although it has been speculated that in medium containing galactose, the amount of capsule produced is higher than compared with glucose (11).

Carbohydrates enter the pneumococcal cell via different transporters, and many of them have been investigated (12). There is complexity to the pneumococcal sugar uptake, as many transporters accept multiple substrates, and many sugars are transported by more than one system (12). The principal glucose transporter in *S. pneumoniae* is the mannose type PTS *manLMN*. It has been shown by mutant analyses that the deletion of this PTS was the only one to affect the utilization of glucose (12) (Fig. 1). However, other routes of glucose importation are hypothesized including uptake by ABC transporters (11). As for fructose, the relevance of the *fruRBA* operon as a phosphoenolpyruvate-phosphotransferase system (PTS) has been described for *S. pneumoniae* but also for Group A Streptococci (12, 13). However, again, the PTS *manLMN* or non-PTS as illustrated for *Escherichia coli* could hypothetically import fructose (12, 14). On the other hand, sucrose (disaccharide glucose-fructose) is probably taken up via a different PTS transporter (*ScrT*) and afterward processed to glucose 6-phosphate and fructose (4).

In this study, we elucidate the influence of three different saccharides (glucose, fructose, and sucrose) on the capsule biosynthesis in various strains of *S. pneumoniae*, both commensal and invasive. For investigating the influence of the different carbon sources on gene expression, RNA-Sequencing (Seq) was conducted and phosphorylated metabolites, which are important for capsule composition were quantified by ^31^P NMR. In addition, isotopologue profiling was performed to study ^13^C-labeled glucose and sucrose incorporation into the pneumococcal capsule and to better understand the mechanism behind the capsule expression.

## Results

### Polysaccharide capsule thickness and growth curves are specific for glucose, fructose, and sucrose

It has been previously hypothesized that exopolysaccharide production is reduced in *S. pneumoniae* in the presence of fructose as the only carbon source (10). Therefore, we first aimed at determining the influence of glucose, fructose, and sucrose (disaccharide glucose-fructose) on capsule thickness. Six wildtype (WT) strains with different serotypes were selected from our collection (Table 1). The structures of the chosen serotypes have been shown and can be found in the supporting material (15) (Table S1).

**Table 1:** ***S. pneumoniae* strains used in this study**

ID	Serotype	Capsule size	RFLP*^a^*	MLST*^b^*
		*bp*		
**Wildtypes**				
106.66	6B	17,506	3	2244
203.24	6C	17,677	11	NA*^c^*
208.41	7F	24,127	8	191
B109.15	7F	24,127	8	191
201.38	9V	20,856	1	644
207.31	15	18,626	1	199
103.57	23F	22,330	11	507
110.58*^d^*	nt	-		344
**Δ*cps* Janus KO mutants**				
106.66 Δ*cps* Janus*^e^*	nt	-	3	2244
208.41 Δ*cps* Janus*^e^*	nt	-	8	191
B109.15 Δ*cps* Janus*^e^*	nt	-	8	191
**Backtransformants**				
106.66 cps 106.66*^e^*	6B	17,506	3	2244
208.41 cps 208.41*^e^*	7F	24,127	8	191
***cps* switch mutants**				
208.41 cps 106.66*^e^*	6B	17,506	8	191
106.66 cps 203.24*^e^*	6C	17,677	3	2,244
106.66 cps 208.41*^e^*	7F	24,127	3	2,244
106.66 cps 201.38*^e^*	9V	20,856	3	2,244
106.66 cps 207.31*^e^*	15	18,626	3	2,244
106.66 cps 103.57*^e^*	23F	22,330	3	2,244

*^a^* Restriction fragment length polymorphism (RFLP) type according to Ref. 9.

*^b^* Multi-locus sequence type (MLST).

*^c^* NA, not applicable.

*^d^* Whole genome sequencing of nt (non-typeable) strain has been described (52).

*^e^* From the collection of strains created by Hathaway *et al.* (9).

**Figure 2.**
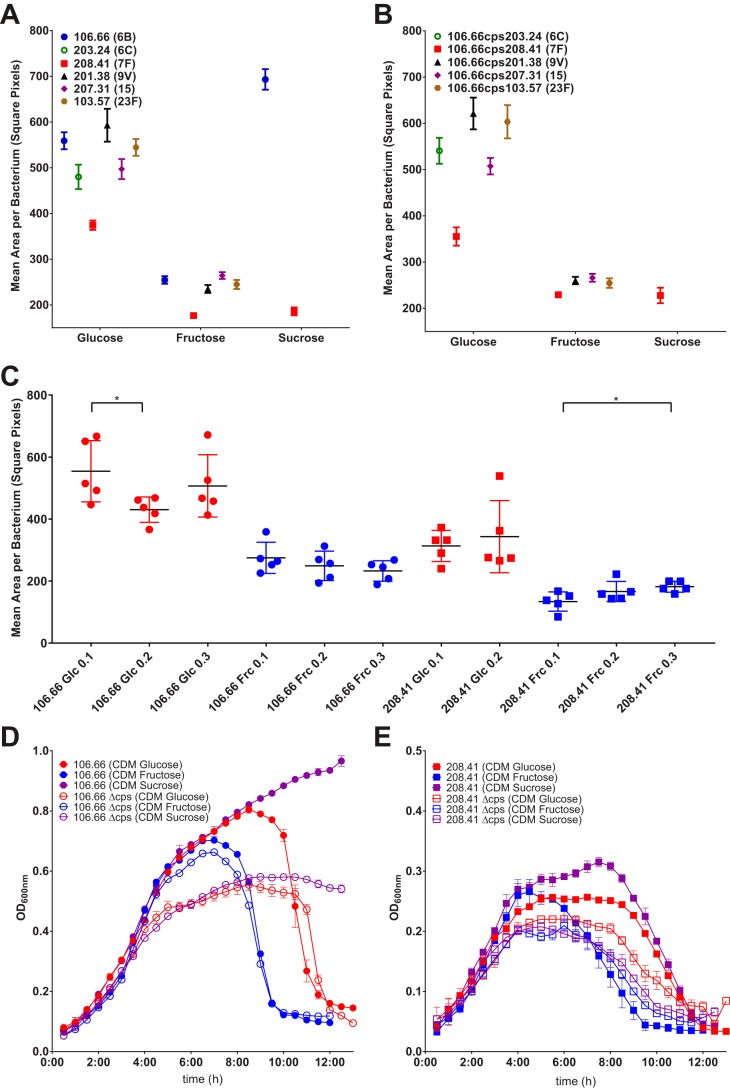
**Capsule thickness from FITC-dextran assay and growth behavior of *S. pneumoniae* in response to changing carbon source availability.** *A,* capsule thickness in WT strains; *B,* capsule thickness of various serotypes on the same genetic background (*cps* switch mutants); *C,* capsule thickness measured during different growth phases, from OD_600 nm_ of 0.1 to 0.3, *D,* growth behavior of strain 106.66 (serotype 6B) and its Δ*cps* KO (knock out) mutant, *E,* growth behavior of strain 208.41 (serotype 7F) and its Δ*cps* KO mutant. As for *A* and *B*, the experiments were done three times on different days and a total of 15 pictures (=15 values) per strain and carbon source were taken.

Capsule thickness was determined by FITC-dextran exclusion assay during mid-log phase of growth in a chemically defined medium (CDM) (Fig. 2). A clear decrease in capsule production was observed for the six chosen strains grown on fructose as compared with glucose-fed strains (Fig. 2*A*). Findings were confirmed in capsule switch mutants with a 106.66 (MLST 2244) background and above mentioned, six serotypes (Fig. 2*B*). In addition, serotype 7F WT (208.41) and cps switch mutant strains (106.66cps208.41) produced the thinnest capsule of all the strains when grown on glucose. We therefore chose 106.66 (a strain with a thick capsule) and 208.41 (a strain with a thin capsule) and their mutants for further experiments. Although capsule thickness was measured at mid-log phase, Fig. 2*C* shows that the growth phase in which the bacteria were harvested did not generally have a large influence on capsule thickness despite two significant findings.

Inspection of the growth curves revealed that whereas bacteria grown on fructose did not reach logarithmic phase any later than when growing on glucose (Fig. 2, *D* and *E*), there was a reduction in the amount of time before the beginning of autolysis. Sucrose on the other hand had the opposite effect, significantly lengthening the time before autolysis in strain 106.66. Both these effects were reduced in the Δ*cps* knockout strains (Fig. 2, *D* and *E*), indicating an effect of the presence of capsular polysaccharide on the longevity of the bacteria. The maximum OD_600 nm_ was significantly different for different serotypes and accordingly, the point of harvest was adjusted to OD_600 nm_ = 0.15 for Serotype 7F strains and OD_600 nm_ = 0.25 for other strains.

**Figure 3.**
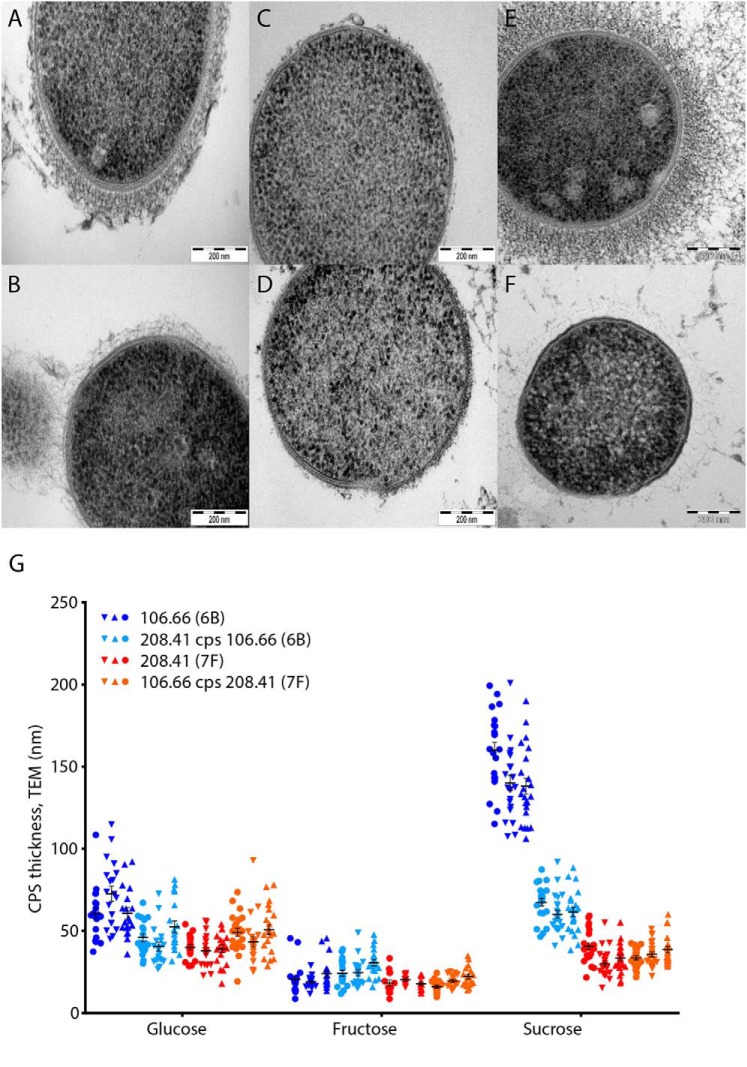
**Capsule thickness measurements perpendicular to the cell wall from TEM images of cells grown on different carbon sources.** Strains 106.66 (*A, C,* and *E*) and 208.41 (*B*, *D*, and *F*) grown on glucose (*A* and *B*), fructose (*C* and *D*) and sucrose (*E* and *F*). Results (*G*) were compiled of between 37 and 67 individual capsule thickness measurements of several individual cells from three separate cultures designated by different *symbols*.

Capsule thickness under the influence of glucose, fructose, and sucrose as the carbon source was also measured by transmission EM for 106.66 and 208.41 wildtypes as well as their *cps* switch mutants (TEM, Fig. 3, *A–G*) and showed a reduction in exopolysaccharide production to almost zero when grown on fructose. Capsule thickness of strains grown on sucrose as a combination of the glucose and fructose monosaccharides was also assessed. The sucrose-fed bacteria were not negatively influenced by the presence of the fructose moiety and produced thick exopolysaccharide capsules. In fact, strain 106.66 WT produced larger amounts of CPS when fed sucrose than when grown on glucose.

### RNA-Seq reveals fructose-specific changes that do not occur in the capsule region

We then performed RNA-Seq of the WT strains 106.66 and 208.41, *cps* mutants, and their back transformants. The total number of paired-reads per sample ranged from 4.79 to 39.65 million paired-reads (average: 20 million reads).

In average 99.03% of reads were mapped onto the pneumococcal genome. Between 54.02 and 93.93% of reads (average: 81.47%) mapped to annotated features. Most of these reads aligned to rRNA. Overall 73.5 or 75.5% of reads aligned to rRNA or tRNA in strain 106.66 or 208.41, respectively.

**Figure 4.**
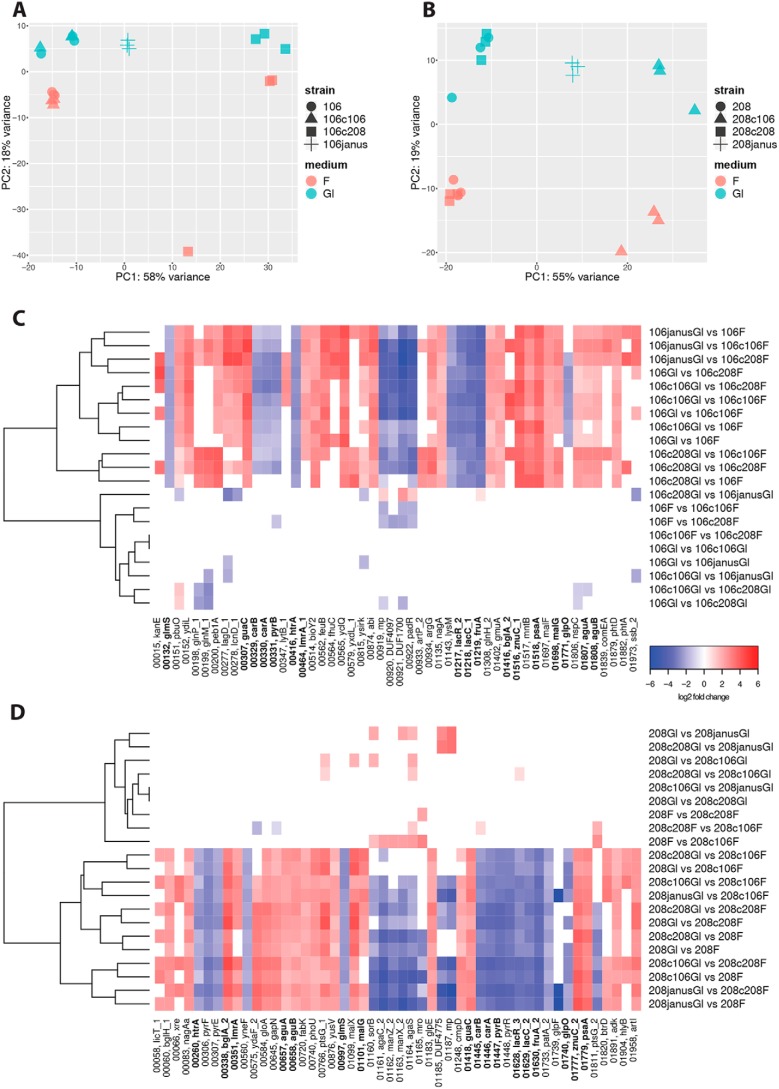
**Principal component analysis and heat maps of RNA-seq data.** Results are visualized according to the strain background 106.66 (MLST 2244; *A*) and 208.41 (MLST 191; *B*). WT and mutant strains were used (see text and Table 1 for more information). Heat maps show the fold-change values for the top 50 genes for each pairwise comparison of the samples according to the strain background 106.66 (*C*) and 208.41 (*D*). Genes that were up- or down-regulated within both are indicated in *bold*. Full gene names and expression values of strains with 106.66 and 208.41 background can be found as Tables S2 and S3, respectively.

The Fig. 4, *A* and *B*, show the principal component analysis (PCA) of all the samples. For the strain 106.66 there is one outlier for one replicate for the mutant with the 7f capsule from strain 208.41. The samples clearly separate by media and capsule for both strains. PCA1 (55–58% of variance) separates data by “capsule,” PCA2 (18–19% of variance) separates data by medium. However, the separation by capsule seems to be driven by expression of genes of capsule region, after removing these genes, there is no separation by capsule in PCA (data not shown).

Fig. 4, *C* and *D,* show the heat maps of the fold-change values for the top 50 genes for each pairwise comparison of the samples. We then more closely inspected genes that were part of or subsequent to the central carbon metabolism of *S. pneumoniae*. In both the 106.66 and the 208.41 strains, we observed that compared to glucose, the *glmS* and *nagA* genes (relevant for peptidoglycan synthesis) are respectively up- and down-regulated in fructose-grown bacteria. The latter was not as significant for 208.41 as in 106.66 but similar log-fold values were received (Tables S2 and S3). RNA-Seq results of *glmS* and *nagA* has been subsequently confirmed with RT-PCR for 106.66 and 208.41 (data not shown). In addition, a number of genes related to PRPP and subsequent pyrimidine synthesis were also increasingly expressed in fructose for both strains (*carA, carB, pyrB*, and *pyrR*). Finally and as expected, fructose transporters fructose transporter genes and Phosphofructokinase (pfk1) were the genes with the highest log-fold differences. Expression values and full names for genes of strains after filtering with 106.66 and 208.41 background can be found as Tables S2 and S3, respectively.

### Analysis of polysaccharide capsule metabolites by ^31^P NMR

**Figure 5.**
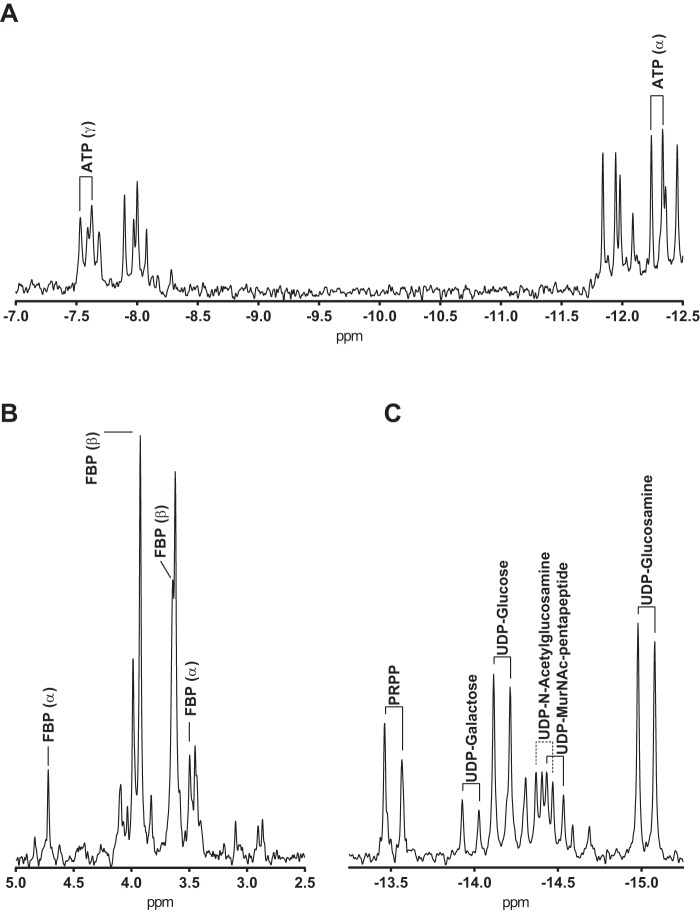
**Excerpt from ^31^P NMR spectra of pneumococcal whole cell ethanol extract (strain 208.41, type 7F).** Metabolite peaks for ATP (*A*) and FBP (*B*) have been identified by spiking. Furthermore, PRPP, UDP-Glc, UDP-Gal, and UDP-GlcNAc have been identified by spiking, whereas UDP-MurNAc-p5 and UDP-GlcN were identified by comparison to previous studies (31) (*C*).

We hypothesized that the lack of capsule polysaccharide in CDM-fructose was due to fructose not being catabolized to the capsule precursors such as UDP-glucose or UDP-galactose (Fig. 1). We therefore compared the intracellular metabolite profiles of *S. pneumoniae* strains grown in CDM supplemented with either glucose, fructose, or sucrose. Because phosphorylated monosaccharide precursors like UDP-glucose and UDP-galactose play a very significant role in capsule biosynthesis (16), we decided to use ^31^P NMR to identify and quantify the phosphorylated metabolites. Fig. 5, *A–C*, show the relevant regions of a whole cell extract ^31^P NMR spectrum of *S. pneumoniae*, including the diphosphodiester region (Fig. 5*C*). UDP-glucose, UDP-galactose, UDP-GlcNAc, ATP, PRPP, and fructose 1,6-bisphosphate (FBP) were identified by addition of pure compounds to the samples (spiking). UDP-glucosamine and UDP-*N*-acetylmuramate-pentapeptide were not available or expensive for spiking and therefore, peaks were assigned by comparison to spectra from previous studies (11, 16). Other precursor molecules like TDP-rhamnose and CDP-choline could not be identified.

**Figure 6.**
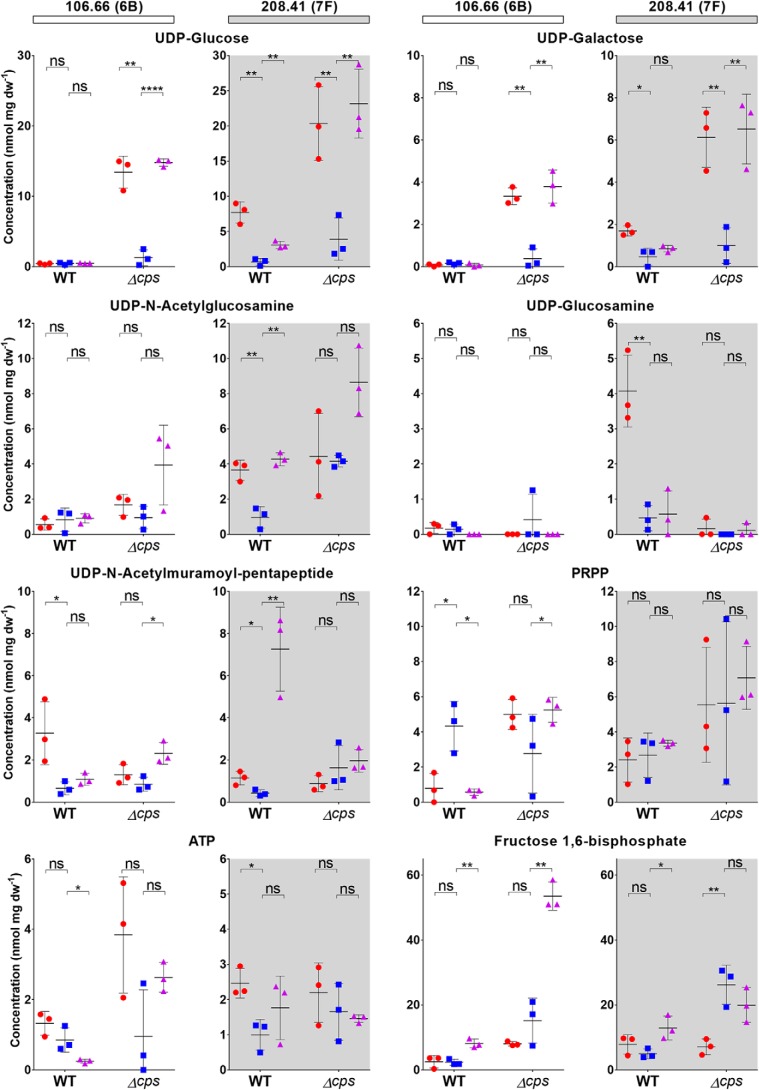
**Metabolite levels observed in *S. pneumoniae* whole cell extracts, biological triplicates from separate cultures.** Overall absolute values were similar to those obtained in a previous study (11). *Red,* CDM-glucose; *blue,* CDM-fructose; *purple,* CDM-sucrose. Differences between conditions were analyzed by unpaired *t* test to determine the significance of results. Significance levels are attributed as not significant (*ns*, *p* > 0.05); *, *p* ≤ 0.05; **, *p* ≤ 0.01; ***, *p* ≤ 0.001; or ****, *p* ≤ 0.0001.

The experiments were then conducted for WT strains and capsule switch mutants (to exclude any effects of the genetic background) as well as Δ*cps* (knockout) strains (Fig. 6 and Fig. S1). We also conducted the experiments using an additional serotype 7F strain (B109.15) and a naturally nonencapsulated strain (110.58) (Table 1 and Fig. S2).

Overall, extracts from Δ*cps* (knockout) strains growing in media supplemented with glucose or sucrose contained a much larger amount of UDP-monosaccharide capsule precursors than their WT counterparts, indicating that the lack of exopolysaccharide biosynthetic mechanism leads to the accumulation of the precursors in the cytoplasm (Fig. 6). In addition, serotype 7F WT isolates accumulated more precursor molecules as compared with 6B strains, which may indicate a less efficient conversion of precursor metabolites into CPS by the exopolysaccharide biosynthetic mechanism. This interpretation is also consistent with the transferability of the effect with the serotype in a *cps* switch mutant (106.66cps208.41), indicating that it is specific to the *cps* operon (Fig. S1). The resulting decreased exopolysaccharide production may explain to some degree the generally thinner capsule of the serotype 7F strains.

Furthermore, Δ*cps* knockout strains growing in CDM supplemented with fructose as the only carbon source produced lower amounts of UDP-glucose and UDP-galactose compared with CDM supplemented with other carbon sources (Fig. 6). This is also true for the naturally nonencapsulated strain but absolute values were lower as compared with Δ*cps* knockout strains (Fig. S2). FBP production, on the other hand, was increased in the presence of fructose as compared with glucose. Another interesting effect was the significant increase of PRPP production in serotype 6B WT bacteria, which is consistent with the observed up-regulation of related genes (*carA, carB, pyrB,* and *pyrR*; Fig. 4). In addition, UDP-*N*-acetylglucosamine was significantly decreased if grown in fructose in WT and capsule switch strains for serotype 7F but not 6B. The combination of both glucose and fructose monosaccharides in a disaccharide (sucrose) resulted in a metabolite pattern resembling a mixture of the two conditions, with the UDP-monosaccharides reaching levels comparable with those reached in CDM-glucose, whereas FBP levels approached or even exceeded those of the fructose-fed bacteria.

### Utilization of fructose subunit of the disaccharide sucrose

**Figure 7.**
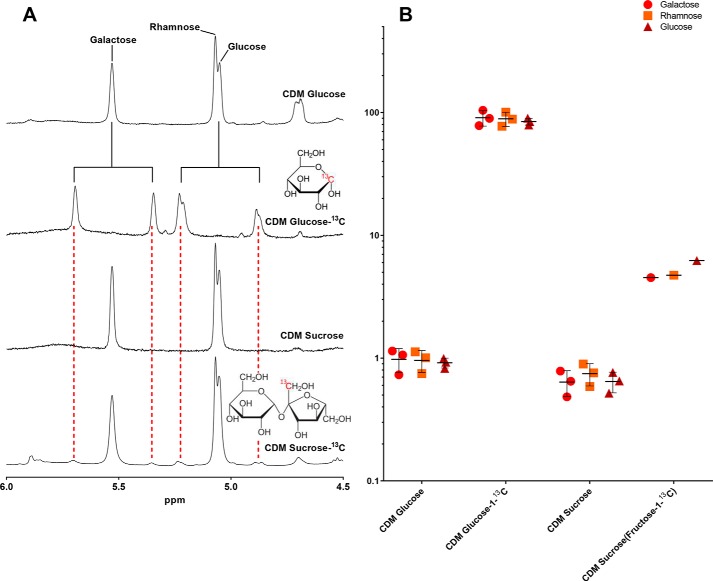
**Results of isotopologue profiling.** *A,* excerpts from ^1^H NMR spectra of serotype 6B capsular polysaccharide showing anomeric proton peaks of galactose, rhamnose, and glucose (repeat unit structure of serotype 6B is shown in Table S1 and is depicted from Ref. 52). Peak splitting due to the heteronuclear coupling between ^1^H and ^13^C nuclei is clearly visible in the extract from pneumococci grown on glucose-1-^13^C. Only minute split peaks are visible in the extract from bacteria grown on sucrose-(fructose-1-^13^C) for identical ppm as compared with glucose-1-^13^C (*red dotted lines*). *B,* quantitative representation of integrals of ^13^C coupling peaks in % of the total capsule extract from three separate cultures under each condition (repeat measurements for sucrose-^13^C were not done due to high pricing of the sugar).

There are several pathways of fructose and sucrose transport and utilization in bacteria (14) (Fig. 1). Because growth of *S. pneumoniae* in CDM-fructose yielded no capsule, but growth in CDM-sucrose resulted in a thick polysaccharide capsule, we hypothesized that the fructose subunit of the disaccharide was mostly metabolized through the glycolysis pathway, allowing the glucose subunit to be catabolized in capsule production. To study the metabolic flux of the fructose subunit of sucrose into the polysaccharide capsule we first cultured the pneumococcal strain 106.66 (serotype 6B) in media containing carbon 13-labeled [^13^C]glucose-1 or sucrose-([^13^C]fructose-1) as the only carbon source. In ^1^H NMR spectroscopy, protons attached to ^13^C display a different peak pattern than protons attached to ^12^C due to the different spin quantum numbers between those two nuclei (Fig. 7, *A* and *B*). Exopolysaccharide from bacteria fed ^13^C-labeled glucose showed distinct and complete splitting of the CPS anomeric proton resonances in ^1^H NMR spectra and strongly increased signals at the relevant chemical shifts in ^13^C NMR spectra. The changes observed in the NMR spectra are characteristic of a complete ^13^C replacement at the relevant positions in the capsule, confirming the incorporation of the labeled monosaccharide into the capsule. The proportion of ^13^C in polysaccharide labeled with [^13^C]glucose was between 81.7 and 91.2% in all three CPS anomeric proton resonances, indicating a very efficient labeling (Fig. 7*B*). Subsequently, we performed an experiment with the labeled fructose moiety of sucrose-([^13^C]fructose-1) and only observed small ^13^C satellite resonances, indicating that its use in capsule biosynthesis is not very efficient (Fig. 7*A*). More specifically, the proportion of ^13^C in polysaccharide labeled on [^13^C]sucrose only contained 4.5% ^13^C for the galactose resonance at 5.53 ppm, 4.7% for the rhamnose resonance at 5.07 ppm, and 6.2% for the glucose resonance at 5.05 ppm (Fig. 7*B*). This finding supports our hypothesis that the polysaccharide capsule is biosynthesized from mostly the glucose subunit of sucrose, whereas the fructose subunit is mostly metabolized through the glycolysis pathway.

## Discussion

The polysaccharide capsule is a major virulence factor and surrounds most clinical strains of the Gram-positive human pathogen *S. pneumoniae*. It functions as a shield protecting underlying bacterial structures from recognition by the immune system, interfering with complement deposition, phagocytosis, and mucus-mediated clearance (17–22). *S. pneumoniae* regulates the thickness of the polysaccharide capsule in response to its environment and this is key in colonization and invasion of epithelial host cells (23). However, despite the importance of the capsule, the mechanisms and environmental conditions that influence capsular polysaccharide expression are not yet very well-understood (23). It has been previously hypothesized that CPS production in *S. pneumoniae* is reduced when grown on fructose as compared with glucose as the carbon source but the mechanism was unclear (10). Within this study, we confirmed that this phenomenon is indeed true for a large number of pneumococcal strains and assessed the mechanism for this phenotypic phenomenon by measuring the intracellular phosphorylated metabolites. In addition, we describe the relevant transcriptional changes from RNA-Seq data if *S. pneumoniae* is grown in fructose as compared with glucose. Finally, we reveal that labeled glucose rather than fructose is incorporated into the capsule if grown in sucrose by stable isotope tracing. Understanding the expression of the capsule in response to the environment is important to future vaccine design.

Pneumococci metabolize carbohydrates such as the monosaccharides fructose, mannose, glucose, galactose, GlcNAc, disaccharides such as sucrose, lactose, and cellobiose, or trisaccharides such as raffinose, which all enter the glycolytic pathway (12). In addition, carbohydrates are involved in the biosynthetic pathways of housekeeping and nonhousekeeping (CPS-specific) components of *S. pneumoniae* CPS (24). Phosphorylated metabolites such as UDP-glucose and UDP-galactose are especially important as precursor molecules for capsule production (11). We therefore focused on quantifying phosphorylated metabolites by ^31^P NMR within this study. This method has been used previously to investigate the effect of *ccpA* deletion on intracellular concentrations of phosphorylated metabolites during growth on glucose or galactose (11). To our knowledge, it has not yet been used to study capsule biosynthesis within *S. pneumoniae.* As compared with HPLC-MS and GC-MS methods for characterizing the pneumococcal metabolome (25), ^31^P NMR only reveals a small subset of (phosphorylated) metabolites. However, ^31^P NMR can distinguish metabolites with identical mass (like the important capsule precursor UDP-glucose *versus* UDP-galactose), which may not be as straightforward with HPLC-MS and GC-MS methods.

By quantifying phosphorylated metabolites, we show that the intracellular concentrations of UDP-glucose and UDP-galactose are drastically decreased in Δ*cps* pneumococcal strains if grown in fructose compared with glucose. This reveals that the pneumococcus is not able to produce the relevant precursors for the biosynthesis of the pneumococcal capsule under fructose conditions. In contrast, exopolysaccharide (EPS) production in *Bifidobacterium longum* has been shown to remain similar when grown on fructose, galactose, and glucose (26). In accordance with our findings, the concentrations of the same two EPS precursors, UDP-glucose and UDP-galactose, were significantly lower in a *Lactococcus lactis* strain containing the EPS gene cluster (Eps+) than in the nonproducer strain MG5267 (Eps−) (16). In a further study focusing on *L. lactis* subsp. *cremoris*, it has been shown that fructose is taken up mainly via the fructose PTS resulting in fructose 1-phosphate (27). In addition, combined actions of 1-phosphofructokinase and FBPase would be required to form essential biomass precursors (27). We hypothesize that the same is true for *S. pneumoniae* due to the following two reasons. First, we revealed that the relevant PTS transporter and *pfk1* are highly up-regulated in fructose from our RNA-Seq data. Considering earlier findings suggesting the presence of *fruA* is essential for growth on fructose (12), we assume that fructose is indeed primarily imported by this PTS transporter. Second, due to the absence of UDP-glucose and UDP-galactose if grown in fructose, FBPase activity seems to be nearly absent in *S. pneumoniae*. However, the product of FBPase, fructose 6-phosphate is essential for polysaccharide capsule production but also peptidoglycan synthesis. Two additional routes for the utilization of fructose have been suggested for *E. coli* (14). Although much less predominant, the additional routes facilitate the production of fructose 6-phosphate. We do not know if they are also the two additional routes for fructose utilization within *S. pneumoniae* at this point. However, we noted an up- and down-regulation of *glmS* and *nagB/nagA*, respectively. These genes are essential for the production of UDP-GlcNAc to build the cell wall, which has been described to be tightly regulated (28, 29). More specifically, *glmS* RNA-specific degradation through the possible participation of a *glmS* riboswitch mechanism has been described (28, 29). Based on our expression results, such degradation might be more abundant if grown on glucose than on fructose, but more detailed experiments will be necessary.

Recently, a mechanism of regulation of the *pyr* operon by the *pyrR* RNA element has been proposed for *S. pneumoniae* (30). It has been shown that in the presence of high UMP but low PRPP, PyrR binds to the *pyrR* RNA and results in the formation of a premature terminator, disrupting the anti-terminator formed when UMP is low (and PRPP high), resulting in transcription termination (30). Within our study, we found both increased intercellular PRPP and expression of genes of the *pyr* operon if grown on fructose. This therefore suggests a tight regulation of this operon as suggested by Warrier *et al.* (30).

Having analyzed the mechanism of CPS biosynthesis and nonbiosynthesis of *S. pneumoniae* grown on glucose and fructose, respectively, we then were wondering how the disaccharide sucrose (fructose-glucose) would be incorporated into the capsule. Sucrose is an important dietary glycan (4) and sucrose metabolism contributes to *in vivo* fitness of *S. pneumoniae* (31). Sucrose is probably imported mainly via a PTS transporters (*ScrT*) and subsequently degraded/hydrolyzed (by *ScrH*) to glucose 6-phosphate and fructose (4). It is hypothesized that a putative fructokinase (*scrK*) produces fructose 6-phosphate (31), which, like glucose 6-phosphate, could be used as substrate for the production of CPS precursor molecules, such as UDP-glucose and UDP-galactose, and, subsequently, capsule biosynthesis (Fig. 1). However, in contrast to labeled glucose, we only noted an incorporation of less than 10% of labeled fructose into the capsule of our serotype 6B strain. More specifically, we received a very reduced labeling of the anomeric protons of rhamnose, galactose, and glucose, which have been previously shown to be characteristic of serotype 6B isolates in NMR spectra (32). Our results are therefore able to quantify the proportion of metabolites used for CPS production within the central carbon metabolism if glucose and fructose monosaccharides are present in equal concentrations within *S. pneumoniae* (as derived from the disaccharide sucrose). We did not consistently detect a decreased production in UDP-*N*-acetylmuramate-pentapeptide in non-EPS producing strains as reported previously (16).

A major strength of our study is the implementation and usage of ^31^P NMR for quantifying phosphorylated (including capsule precursor) metabolites. Doing this for strains grown on glucose as compared with fructose, we were able to provide mechanistic evidence for the absence of the capsule in the latter condition. By additionally performing RNA-Seq, we were also able to reveal relevant gene expression differences. Another particular strength of our study is the usage of isotopologue profiling, which offers the possibility to decipher the incorporation of the distinct carbon sources into the capsule. This may seem less important for monosaccharides such as glucose (as incorporation of ^13^C-labeled glucose is nearly 100%) but, in contrast, is very relevant for di- and oligosaccharides (such as sucrose), as the proportion of utilization of the different monosaccharides in CPS biosynthesis is unknown.

A limitation of the study is the fact that we did not yet check the influence of larger fructooligosaccharides (FOS) on the expression of the capsule. FOS are taken up by different (ABC) transporters (4, 33) and, once imported, are thought to be degraded by *FusH* (4). The exact products of this enzymatic reaction and their effects on CPS biosynthesis are unknown. Also, we did not investigate the effects of other monosaccharides, such as galactose, mannose, and GlcNAc on CPS production, as our main aim in this study was to elucidate the cause of the absence of CPS in strains grown on fructose. It is known that pneumococcal strains without capsule are more prone to opsonophagocytosis and, therefore, it remains to be analyzed if increased concentrations of fructose and/or FOS could potentially be used as prebiotic for preventing pneumococcal colonization and/or subsequent invasive disease.

We did not detect UDP-GalNac in the ^31^P spectra of the strains with 7F serotype, which was also not reported for pneumococcal strain D39 in a recent study (11). This is perhaps surprising considering that the serotype 7F strains possess GalNac within the capsule structure (Table S1) but the building of UDP-GalNac is probably important for all pneumococcal strains for the biosynthesis of teichoic acids (34). We may only speculate if UDP-GalNAc is generally consumed very rapidly in *S. pneumoniae* and therefore difficult to detect using the methods presented.

However, we observed a less efficient conversion of precursor metabolites into the CPS in 7F. The accumulation of UDP-precursors could be the result of a relative bottle-neck step in epimerization done by the GalE enzyme (Fig. 1), which has been shown to be able to epimerize both, UDP-glucose/UDP-galactose, and UDP-GlcNAc-UDP-Gal-NAc in *S. pneumoniae* (35).

In summary, this study reveals and quantifies the dual utilization of carbohydrates for capsule production and glycolysis. It further shows that some serotypes (*e.g.* 6B) are more efficient in the conversion of precursor molecules to capsular polysaccharide than others (*e.g.* 7F). Understanding the metabolic flux of different carbohydrates and their relationship to capsule expression is important considering the wide range of host-derived glycans the pneumococcus encounters during its interaction in the human host (4, 7). Knowledge about the pneumococcal metabolism and mechanism of capsule biosynthesis is also important to recognize potential new drug targets.

## Experimental procedures

### Bacterial strains and growth conditions

The bacterial strains for this study were selected from the collection of the Swiss National Reference Centre for Pneumococci. Strains for the preliminary experiments were chosen to include a variety of serotypes. This included strains of serotypes 6B, 6C, 7F, 9V, 15, and 23F and their capsule switch mutants. The structures of the serotypes have been shown and can be found in the supporting material (15) (Table S1). For the more detailed analyses, strains 106.66 (serotype 6B) and 208.41 (serotype 7F) were chosen as examples of serotypes more commonly associated with commensal colonization of the nasopharynx and invasive disease, respectively, as well as representing one strain with a thick polysaccharide capsule and one with a thin capsule (Table 1). Bacteria were cultured as described previously (36). Briefly, bacteria were streaked out on CSBA plates and grown for ∼10 h at 37 °C in a 5% CO_2_ atmosphere. They were then inoculated into tubes containing modified Lacks medium (37–39) supplemented with glucose and grown to an OD_600 nm_ of 0.5. After centrifugation and washing, 3 ml of bacterial suspension at OD_600 nm_ 0.5 were added to 150 ml of CDM supplemented with a single carbon source at a concentration of 5.5 mm (5.5 mm glucose is approximately naturally found in the blood). For the isotopologue profiling experiments, CDM containing 5.5 mm of either 100% labeled or unlabeled carbohydrates were used. The cultures were then grown further to mid-logarithmic phase. Bacterial growth was tracked by measuring the optical density at a wavelength of 600 nm (OD_600 nm_) using a Thermo Scientific Helios Epsilon UV-visible spectrophotometer with an adapter to allow measurement of OD_600 nm_ directly in the culture tubes.

### FITC dextran capsule thickness measurements

Capsule thickness was evaluated in biological triplicate by fluorescence microscopy according to a method previously described (10, 40, 41). Briefly, cells were harvested by centrifugation at mid-log phase, washed, and resuspended. Cells were prepared for visualization on a microscope slide and imaged as described before (9). At least 5 brightfield and fluorescence images per sample were recorded and the area of fluorescence occlusion per cell was calculated using free ImageJ software as previously described (40). The experiments were done three times on different days and a total of 15 pictures (=15 values) per strains and carbon source were taken.

### TEM imaging

Cells were grown using the methods described above and harvested at mid-log phase. Samples for TEM imaging were prepared as described before (40, 42). In brief, bacteria were pelleted by centrifugation (5000 rpm for 5 min) and the supernatant was discarded. Bacteria were then cryopreserved by high-pressure freezing (HPF) as described by Studer *et al.* (42) using 1.4 × 0.1-mm membrane carriers (Leica Microsystems, Vienna) coated with l-α-phosphatidylcholine (Fluka, Buchs, Switzerland). Acetone containing 2% osmium tetroxide, 0.1% uranyl acetate, 0.2% ruthenium hexamine trichloride and a total of 4% H_2_O served as the medium for freeze substitution. The ruthenium hexamine trichloride was added to improve capsule resolution (43). After substitution, bacteria were washed in acetone (Merck, Darmstadt, Germany) four times for 30 min each, they were then incubated for 2 h and 30 min in acetone:Epon (2:1) and 4 h in acetone:Epon (1:1) followed by an overnight incubation with acetone:Epon (1:2) at room temperature. The next day, samples were embedded in Epon (Sigma) and left to harden at 60 °C for 5 days. Ultrathin sections (75 nm) were produced with an ultramicrotome UC6 (Leica Microsystems, Vienna, Austria). The sections, mounted on Formvar® (Ted Pella Inc. USA)-coated single slot copper grids, were stained with uranyl acetate (Electron Microscopy Sciences, Hatfield, PA) and lead citrate (Leica Microsystems, Vienna, Austria) with an ultrostainer (Leica Microsystems). Sections were examined with a transmission electron microscope (CM12, Philips, Eindhoven) equipped with a digital camera (Morada, Soft Imaging System, Münster, Germany) and image analysis software (iTEM). Measurements of capsule thickness were conducted on several images and several points on cell bodies perpendicular to the pneumococcal cell wall using the software ImageJ. For each strain and carbon source, a total of between 37 and 67 measurements were conducted.

### Whole genome and RNA-Seq of WT and mutant strains

We first sequenced the reference genomes (106.66 and 208.41) by Illumina HiSeq 2000 (Illumina Inc.). The resulting reads were *de novo* assembled using SPAdes (44). Gene prediction was performed with Prodigal (45) and annotation was a carry-over from strain 23F. The assembled genomes are available under PRJNA554545.

Overall for each strain 7 samples each in 3 replicates were analyzed with RNA-sequencing with the accession number PRJNA554543. The 7 samples were the original strain, a mutant with transformed capsule from the other stain and a mutant with the back transformed capsule in two media; with fructose or glucose; and a mutant without a capsule in medium with glucose. RNA libraries were sequenced as paired-end reads on Illumina. Reads from RNA-sequencing were mapped to the reference genomes with bowtie2 (46, 47) and following counting of the reads was performed with featureCounts (48).

Genes annotated as rRNA, tRNA, transposases, and manually confirmed hypothetical proteins were excluded from the analysis. After this step 2095 and 1944 genes from 2163 and 1997 genes were left for strains 106.66 and 208.41, respectively. Reads were then normalized as a regularized log (rlog), a data transformation method in DESeq2 (49), and as transcripts per million (TPM) (50). Low expression genes were categorized from TPM values. Decile values were used to partition expression values of genes into 10 classes. The first decile was used as the maximum limit for low expressed genes. 2.1 TPM for strain 106.66 and 2.9 TPM for strain 208.41 were set as the minimum expression value, 1966 for strain 106.66 and 1829 genes for strain 208.41 were left for further analysis. The very short genes were also removed from the analysis. The same parameters as for low expressed genes were used: the first decile was used to define the short genes. With these criteria all genes shorter than 225 bases (strain 106.66) or 240 bases (strain 208.41) were removed from further analysis. Overall 145 genes in strain 106.66 and 148 genes in strain 208.41 were removed. After all filtering 86% of genes were left for the analysis (1821 of2163 genes for strain 106.66, and 1681 of1997 genes for strain 208.41) (Tables S2 and S3).

Fold-changes were calculated for each pair of conditions, resulting in a total of 21 comparisons. Fold-changes of genes for which significance could not be calculated confidently, defined as adjusted *p* value greater than 0.01, were set to 0. Then the fold-changes were calculated over all conditions comparing the values in glucose and fructose media. The fold-changes of genes with an adjusted *p* value bigger than 0.01 were set to 0. The ranking was made based on this comparison. For the ranking, the absolute value of the fold-change was taken. The capsule genes were analyzed separately. The fold-changes of genes for which none of the pairwise comparisons was significant were also set to 0. Overall 737 genes for strain 106.66 and 756 genes for strain 208.41 had significant fold-changes and 41 genes for strain 106.66 and 47 genes for strain 208.41 had fold-changes bigger than 2. The top 50 genes with the highest absolute fold-change were used for further analysis.

### Intracellular metabolite extraction

Whole cell EtOH extracts were prepared using a slight modification of the method of Ramos *et al.* (16). In brief, the bacteria cultured in chemically defined medium were harvested by centrifugation and washed once each with ice-cold 0.8% NaCl and ice-cold H_2_O. They were then resuspended in ice-cold H_2_O and diluted with absolute EtOH at −20 °C to a concentration of 60% EtOH. Cells were disrupted via vigorous shaking for 2 h at 0 °C. Cell debris was removed by ultracentrifugation and ultrafiltration (Amicon 10-kDa centrifuge filter) and the solvent was evaporated under reduced pressure. Dried samples were weighed and dissolved in 100 μl of NMR buffer (20 mm MOPS, 5 mm NaOAc, and 1 mm EDTA in D_2_O with 0.1% phosphonoacetic acid (PPA), and 0.1% TSP, pH 7.4) transferred into 1.7-mm NMR microtubes and submitted for measurement of ^31^P NMR spectra.

### Capsular polysaccharide extraction and analysis

Extracts of the capsular polysaccharide were obtained as described previously (9, 36). For isotopologue profiling experiments, the pneumococci were provided a single carbon source, either unlabeled or labeled with ^13^C in the 1-position at 100% concentration (5.5 mm). For serotype analysis, regular unlabeled saccharides were used. Bacterial cultures were grown as described above, harvested by centrifugation, and washed with ice-cold H_2_O. After resuspension, capsule polysaccharide was separated from the cells by addition of buffer-saturated phenol to a concentration of 1% and incubation overnight at room temperature. Cell debris was removed by centrifugation and nucleotides and peptides were digested by addition of nuclease and proteinase K, respectively. CPS was separated from smaller molecules using Millipore Amicon Ultra 30-kDa cut off membrane centrifugal filter units and the solvent was removed under reduced pressure. Dried capsule polysaccharide samples were dissolved in 100 μl of D_2_O transferred into 1.7-mm NMR microtubes and submitted for NMR measurements.

### Metabolic flux analysis by isotopologue profiling

Metabolic flux analysis of carbohydrate uptake was based on the principle of ^13^C-based isotopologue profiling (51). Bacteria were grown as described above using ^13^C-labeled monosaccharides as the carbon source. In particular, [^13^C]glucose-1 was used to create completely labeled capsular polysaccharides as a reference point. To track the metabolic flux of different monosaccharides, sucrose with an unlabeled glucose subunit and a labeled fructose 1-^13^C subunit was used. By extracting the capsule and analyzing it by NMR as described, it was possible to determine the proportions of labeled and unlabeled saccharides in the polysaccharide capsule.

### NMR measurements

NMR data were collected on a Bruker Avance II (500 MHz; ^1^H) spectrometer equipped with a 1.7-mm triple-resonance (^1^H, ^13^C, ^31^P) microprobe head. The samples were prepared as follows. The full amount of each capsule extract (∼5–10 mg) was dissolved in 100 μl of NMR buffer (MOPS/EDTA in D_2_O with 0.1% PPA and 0.1% TSP, pH 7.4) or pure D_2_O and 65 μl of the resulting mixtures were transferred into 1.7-mm NMR tubes. ^31^P spectra were acquired using 4096 scans with a spectral width of 40760.9 Hz, a recycling delay of 2 s, and an acquisition time of 0.402 s. ^1^H spectra were recorded using 1024 scans with a spectral width of 12500.0 Hz, a recycling delay of 1 s, and an acquisition time of 1.311 s. All spectra were acquired at a regulated temperature of 298 K. All experiments were recorded using the TopSpin® software, version 3.2 (Bruker Biospin), and processed using TopSpin® version 4.0.5. For the intracellular metabolite extracts, one-dimensional ^31^P NMR was used, whereas for the capsule extracts, one-dimensional ^1^H NMR as well as two-dimensional ^1^H/^13^C-heteronuclear single quantum coherence spectra were recorded.

### Identification and quantification of metabolite signals

^31^P NMR resonances were assigned to metabolites by spiking samples with specific metabolites in known concentrations and comparison to previous studies. Quantification of the metabolites was achieved by comparison to the quantity of the internal PPA standard following a classical calibration procedure. A specific conversion factor was obtained from this calibration for each metabolite and was used to calculate absolute amounts from the NMR integrals of the metabolites and PPA. To ensure consistency of the results, all metabolite extract samples were dissolved in NMR buffer from the same batch. Metabolite concentration data were analyzed by unpaired *t* test to determine the significance of results.

## Author contributions

L. J. T., J. F., and M. H. data curation; L. J. T., J. P. W., T. O. S., N. M., C. S., S. D. B., and M. H. investigation; L. J. T., N. M., and J. F. visualization; L. J. T., J. P. W., T. O. S., P. V., M. V., D. W., C. S., R. B., L. J. H., J. F., and M. H. methodology; L. J. T. writing-original draft; N. M., D. W., and C. S. software; P. V., M. V., D. W., S. D. B., L. J. H., and J. F. writing-review and editing; C. S. resources; S. D. B. and M. H. conceptualization; L. J. H. and M. H. supervision; M. H. validation; M. H. project administration.

## Supplementary Material

Supporting Information

## Footnotes

2 The abbreviations used are:

CDM: chemically defined medium
CPS: capsular polysaccharide
FBP: fructose 1,6-bisphosphate
FITC: fluorescein isothiocyanate
FOS: fructooligosaccharides
GlcA: glucuronic acid
Glc-N: glucosamine
MurNAc-p5: *N*-acetylmuramate-pentapeptide
PRPP: phosphoribosyl pyrophosphate
PTS: phosphotransferase transport system
TEM: transmission electron microscopy
UDP: uridine diphosphate
PCA: principal component analysis
EPS: exopolysaccharide
TPM: transcripts per million
PPA: phosphonoacetic acid
nt: nucleotide.
