# Supplementary material for: Carbon source regulates polysaccharide capsule biosynthesis in Streptococcus pneumoniae
Source: J Biol Chem. 2019 Oct 8;294(46):17224–38. doi: 10.1074/jbc.RA119.010764 (PMC6873171; doi:10.1074/jbc.RA119.010764)
Supplement: Supporting Information [file supp_294_46_17224__index.html]

Carbon source regulates polysaccharide capsule biosynthesis in Streptococcus pneumoniae — Mechanisms for the synthesis of the pneumococcal capsule — Carbon source regulates polysaccharide capsule biosynthesis in Streptococcus pneumoniae — Mechanisms for the synthesis of the pneumococcal capsule — Supporting Information 

# Carbon source regulates polysaccharide capsule biosynthesis in *Streptococcus pneumoniae*

## Supporting Information

- Supporting Information (to be published online) - Figure s1
- Supporting Information (to be published online) - Figure s2
- Supporting Information (to be published online) - Supplementary table 1
- Supporting Information (to be published online) - Supplementary table 2
- Supporting Information (to be published online) - Supplementary table 3
