# Supplementary material for: Carbon source regulates polysaccharide capsule biosynthesis in Streptococcus pneumoniae
Source: J Biol Chem. 2019 Oct 8;294(46):17224–38. doi: 10.1074/jbc.RA119.010764 (PMC6873171; doi:10.1074/jbc.RA119.010764)
Supplement: Supporting Information [file supp_RA119.010764_155365_1_supp_399075_pz0fz4.pdf]

Figure S1: Metabolite levels observed in *S. pneumoniae cps* switch whole cell extracts, biological triplicates. Overall values were similar to those obtained in previous studies (11) and metabolite profiles of *cps* switch mutants are similar to the wild types of the same serotype. (Red) CDM-glucose; (Blue) CDM-fructose; (Purple) CDM-sucrose. Differences between conditions were analyzed by unpaired t-test in order to determine the significance of results. Significance levels are attributed as not significant (ns, $p>0.05$), * ($p\leq0.05$), ** ($p\leq0.01$), *** ($p\leq0.001$) or **** ($p\leq0.0001$).

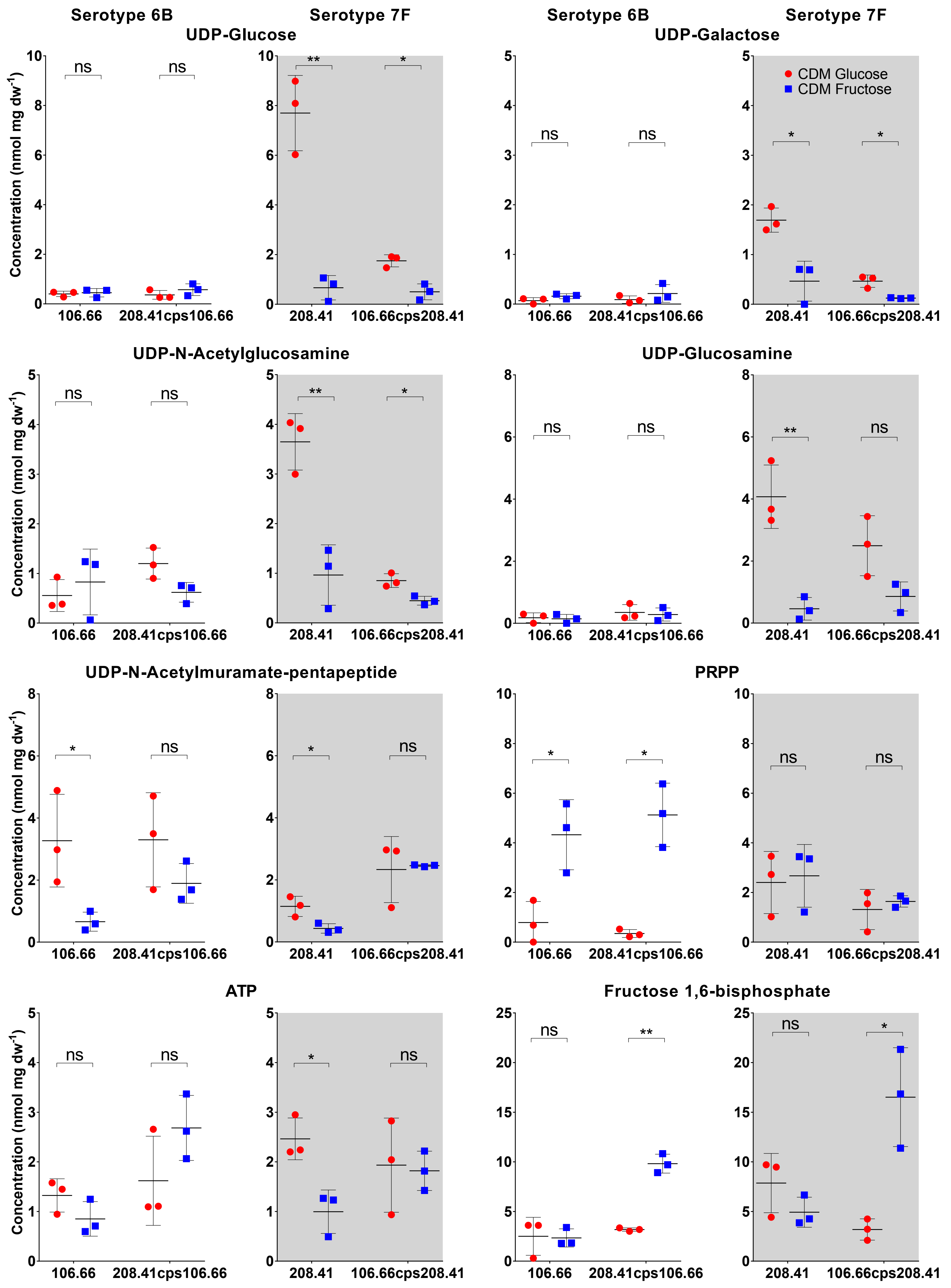

Serotype 6B
Serotype 7F
Serotype 6B
Serotype 7F
UDP-Glucose
UDP-Galactose
CDM Glucose
CDM Fructose
Concentration (nmol mg dw^{-1})
ns
*
**
106.66
208.41
208.41cps106.66
106.66cps208.41
UDP-N-Acetylglucosamine
UDP-Glucosamine
UDP-N-Acetylmuramate-pentapeptide
PRPP
ATP
Fructose 1,6-bisphosphate
