# Supplementary material for: Carbon source regulates polysaccharide capsule biosynthesis in Streptococcus pneumoniae
Source: J Biol Chem. 2019 Oct 8;294(46):17224–38. doi: 10.1074/jbc.RA119.010764 (PMC6873171; doi:10.1074/jbc.RA119.010764)
Supplement: Supporting Information [file supp_RA119.010764_155365_1_supp_399076_pz0fz4.pdf]

Figure S2: Metabolite levels observed in *S. pneumoniae* strains B109.15 (ST 7F) and 110.58 (nontypeable) whole cell extracts, biological triplicates. Overall values were similar to those obtained in previous studies (11). B109.15 metabolite profiles are similar to those observed in other serotype 7F strains. (Red) CDM-glucose; (Blue) CDM-fructose. Differences between conditions were analyzed by unpaired t-test in order to determine the significance of results. Significance levels are attributed as not significant (ns, $p>0.05$), * ($p\leq0.05$), ** ($p\leq0.01$), *** ($p\leq0.001$) or **** ($p\leq0.0001$).

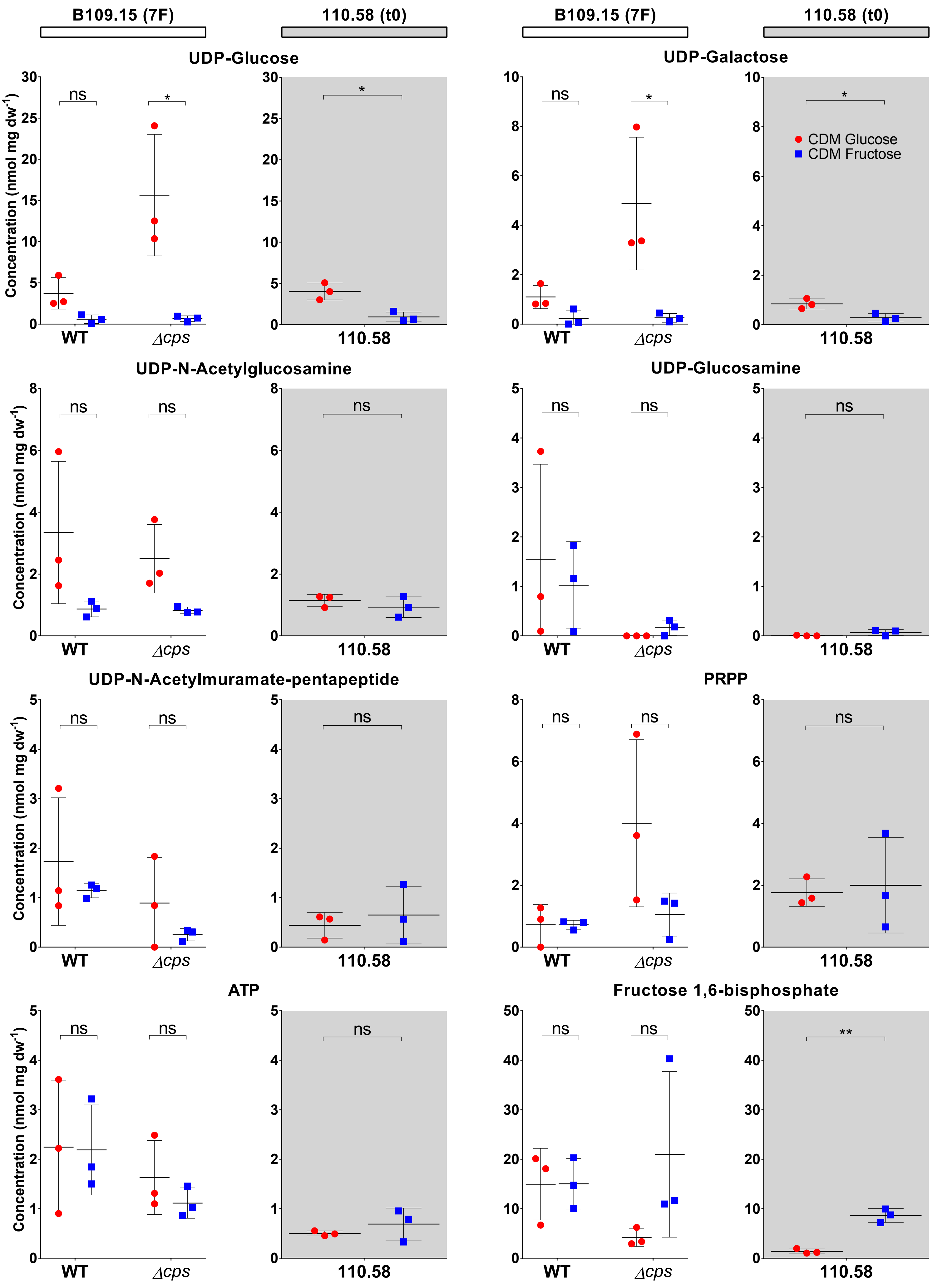
B109.15 (7F)
110.58 (t0)
B109.15 (7F)
110.58 (t0)
UDP-Glucose
UDP-Galactose
UDP-N-Acetylglucosamine
UDP-Glucosamine
UDP-N-Acetylmuramate-pentapeptide
PRPP
ATP
Fructose 1,6-bisphosphate
Concentration (nmol mg dw^{-1})
CDM Glucose
CDM Fructose
ns
*
**
WT
Δcps
110.58
