# Supplementary material for: Carbon source regulates polysaccharide capsule biosynthesis in Streptococcus pneumoniae
Source: J Biol Chem. 2019 Oct 8;294(46):17224–38. doi: 10.1074/jbc.RA119.010764 (PMC6873171; doi:10.1074/jbc.RA119.010764)
Supplement: Supporting Information [file supp_RA119.010764_155365_1_supp_404459_pyqxbh.docx]

| **Serotype** | Structure |
| --- | --- |
| **6B** | 🡪2)-α-D-gal*p*-(1🡪3)-α-D-glc*p*-(1🡪3)-α-L-rha*p*-(1🡪4)-D-rib-ol-(5🡪P |
| **6C** | 🡪2)-α-D-glc*p*-(1🡪3)-α-D-glc*p*-(1🡪3)-α-L-rha*p*-(1🡪3)-D-rib-ol-(5🡪P |
| **7F** | 🡪6)-α-D-gal*p*-(1🡪3)-β-L-rha*p*2Ac-(1🡪4)-β-D-glc*p*-(1🡪3)-β-D-gal*p*NAc-(1🡪  2 4  ↑ ↑  1 1  β-D-gal*p* α-D-glc*p*NAc-(1🡪2)-α-L-rha*p* |
| **9V** | 🡪4)-α-D-glc*p*A2_0.25_,3_0.55_Ac_2_-(1🡪3)-α-D-gal*p*-(1🡪3)-β-D-man*p*NAc4_0.09_,6_1.04_Ac_2_-(1🡪4)-β-D-glc*p*-(1🡪4)-α-D-glc*p*-(1🡪 |
| **15C** | 🡪6)-β-D-glc*p*NAc-(1🡪3)-β-D-gal*p*-(1🡪4)-β-D-glc*p*-(1🡪  4  ↑  1  α-D-gal*p* –(1🡪2)-β-D-gal*p*  3  ↑  Gro_0.7_-(2🡪P |
| **23F** | Gro-(2🡪P  ↓  3  🡪4)-β-D-glc*p*-(1🡪4)-β-D-gal*p*-(1🡪4)-β-L-rha*p*-(1🡪  2  ↑  1  α-L-rha*p* |

Supplementary table 1: Biochemical structures of serotypes included in the study
